# Novel Food Processing and Extraction Technologies of High-Added Value Compounds from Plant Materials

**DOI:** 10.3390/foods7070106

**Published:** 2018-07-05

**Authors:** Predrag Putnik, Jose M. Lorenzo, Francisco J. Barba, Shahin Roohinejad, Anet Režek Jambrak, Daniel Granato, Domenico Montesano, Danijela Bursać Kovačević

**Affiliations:** 1 Faculty of Food Technology and Biotechnology, University of Zagreb, Pierottijeva 6, 10000 Zagreb, Croatia; arezek@pbf.hr; 2 Centro Tecnológico de la Carne de Galicia, rúa Galicia 4, Parque Tecnológico de Galicia, San Cibrao das Viñas, 32900 Ourense, Spain; jmlorenzo@ceteca.net; 3 Nutrition and Food Science Area, Preventive Medicine and Public Health, Food Sciences, Toxicology and Forensic Medicine Department, Faculty of Pharmacy, Universitat de València, Avda. Vicent Andrés Estellés, s/n, 46100 Burjassot, València, Spain; Francisco.Barba@uv.es; 4 Department of Food Science and Nutrition, University of Minnesota, St. Paul, MN 55108, USA; sroohine@umn.edu; 5 Burn and Wound Healing Research Center, Division of Food and Nutrition, Shiraz University of Medical Sciences, Shiraz 71348-14336, Iran; 6 Department of Food Engineering, State University of Ponta Grossa. Av. Carlos Cavalcanti, 4748, 84030-900 Ponta Grossa, Brazil; granatod@gmail.com; 7 Department of Pharmaceutical Sciences, Section of Food Science and Nutrition, University of Perugia, Via San Costanzo 1, 06126 Perugia, Italy; domenico.montesano@unipg.it

**Keywords:** functional food, extract, biological active compounds, innovative technology

## Abstract

Some functional foods contain biologically active compounds (BAC) that can be derived from various biological sources (fruits, vegetables, medicinal plants, wastes, and by-products). Global food markets demand foods from plant materials that are “safe”, “fresh”, “natural”, and with “nutritional value” while processed in sustainable ways. Functional foods commonly incorporate some plant extract(s) rich with BACs produced by conventional extraction. This approach implies negative thermal influences on extraction yield and quality with a large expenditure of organic solvents and energy. On the other hand, sustainable extractions, such as microwave-assisted extraction (MAE), ultrasound-assisted extraction (UAE), high-pressure assisted extraction (HPAE), high voltage electric discharges assisted extraction (HVED), pulsed electric fields assisted extraction (PEF), supercritical fluids extraction (SFE), and others are aligned with the “green” concepts and able to provide raw materials on industrial scale with optimal expenditure of energy and chemicals. This review provides an overview of relevant innovative food processing and extraction technologies applied to various plant matrices as raw materials for functional foods production.

## 1. Introduction

**Figure 1:**
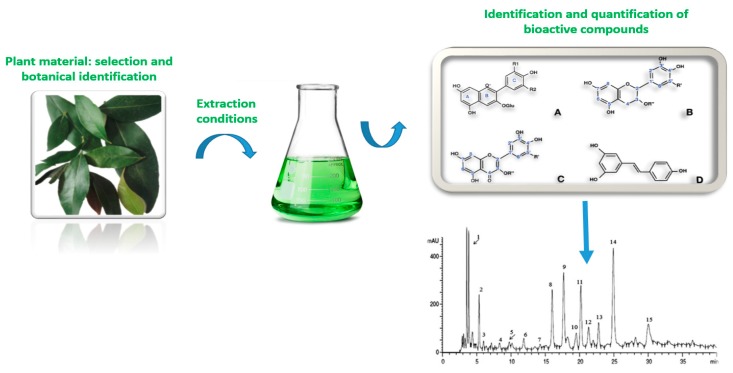
Bioactive compounds from plant extracts: from selection of plant materials to quantification of BACs.

**Figure 2:**
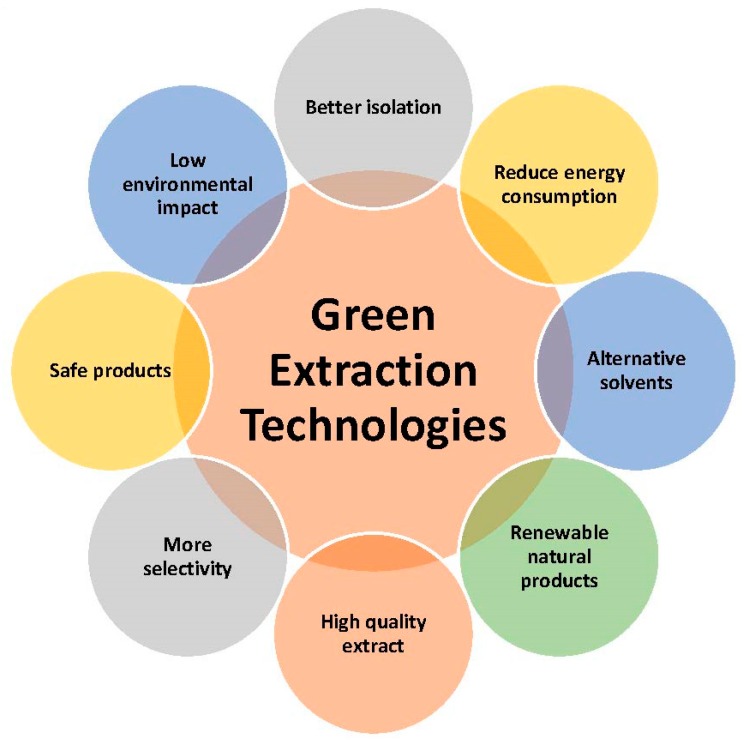
Advantages of the use of green technologies to extract bioactive compounds from plant sources.

Usually, functional foods are considered as food product identical in all aspects to conventional foods except that it contains some biologically active compound (BAC) as an added ingredient [1]. Even though designing a new food product is generally an expensive venture [2], functional foods can be developed practically from various raw materials by means of the process of enrichment, fortification or other alteration of nutritive components [3]. Recent trends in global food markets showed that consumers demand foods from plant materials that are deemed as “safe”, “fresh”, “natural”, and with “nutritional value” while produced and processed with sustainable methods [4]. Such raw materials include minimally processed fresh fruits [5,6,7,8,9,10,11,12,13,14], vegetables [15,16], medicinal and aromatic plants [17,18,19], and their wastes and by-products [4,20,21,22,23]. Currently, functional foods commonly incorporate some plant extract(s) rich in BACs (Figure 1) that are produced with conventional extraction that frequently has some negative thermal influences on extraction yield and quality [24]. Hence, it is preferable to obtain extracts by some sustainable extractions, such as microwave-assisted extraction (MAE), ultrasound-assisted extraction (UAE), high-pressure assisted extraction (HPAE), high voltage electric discharges assisted extraction (HVED), pulsed electric fields assisted extraction (PEF), supercritical fluids extraction (SFE), among other methods [17,25,26]. These techniques are aligned with the “green” concepts (Figure 2) and able to provide raw materials on the industrial scale with optimal expenditure of energy and chemical solvents [27,28]. Engineered food products should have acceptable food structure, composition, and stability that is supportive of their traceability and authenticity [29]. The main objective of this review is to address the topics relevant to novel food processing and extraction technologies applied to various plant matrices as raw materials for functional foods production.

## 2. Health Benefits of Plant Materials

As earlier mentioned, health benefits in functional foods frequently come from BACs and depend on their extraction (natural) sources and the chemical structure. Plants have different mixtures of BACs that exert medicinal benefits; however, generally they have a single characteristic group of compounds that provide typical medicinal roles in the human body. For instance, antioxidative polyphenols from apple (*Malus domestica* L.) are phenolic acids, e.g., chlorogenic and coumaroylquinic acids, and flavonoids, e.g., epicatechin, phloretin, and quercetin [30]. The flesh of mandarin fruit (*Citrus reticulate* Blanco) contains high nutritive content of antioxidants, (e.g., ascorbic acid, carotenoids, and phenolic compounds), in addition to sugars, organic acids, amino acids, pectin, minerals, and volatile organic compounds. Additionally, peel contains essential oils rich with limonene, β-myrcene, 3-carene, and α-pinene [13]. The most of the health benefits associated with mandarin fruits come from antioxidative activity of its BACs. Next, the prickly pear fruit (*Opuntia* spp.) contains high contents of polyphenols, betalains, betacyanins, ascorbic acid, amino acids, minerals, and other compounds [31,32,33,34,35] that are associated with antioxidant, antiatherogenic, antiulcerogenic health benefits, as well as prevention of low-density lipoproteins peroxidation [36,37,38]. Berry fruits such as bearberry (*Arctostaphylos* sp.), blueberry (*Vaccinium* sp.), blackberry (*Rubus* sp.), blackcurrant (*Ribes nigrum*), cranberry (*Vaccinium* sp.), cloudberry (*Rubus chamaemorus*), strawberry (*Fragaria ananassa*), pomegranate (*Punica granatum*), and grape berries (*Vitis* sp.), and their extracts are rich in antioxidants [18].

Mainly this is referred to the ascorbic acid and polyphenols (e.g., anthocyanins, phenolic acids, flavanols, flavonols, and tannins), with reported health benefits for cardiovascular diseases, cancer, rheumatoid arthritis, lung diseases, cataract, Parkinson’s, and Alzheimer’s disease [14,18,39]. One of the important vegetable representatives are *Allium* sp. (e.g., garlic, onion, leeks, and chives) that are known for their organosulfur compounds (OSC) even though they contain high quantities of flavonoids, steroidal saponins, and phytosterols [40]. Their multiple health benefits include antimicrobial, antiviral, antidiabetic, anti-protozoal, antioxidant, antispasmodic, anticarcinogenic, antimutagenic, antiasthmatic, anti-amnesic, anti-inflammatory, hepatoprotective, neuroprotective, hypotensive, hypoglycemic, immunomodulatory, urease/xanthine oxidase inhibitors, and prebiotic properties [41,42,43,44,45,46]. Pumpkin (*Cucurbita* sp.) is another vegetable that has considerable amounts of BACS in a form of terpenoids (especially carotenoids) responsible for the improvement of the immune system, lower the risk of cardiac and cancer diseases and enlargement of prostate gland [15]. Some recognized sources of BACs are rosemary (*Rosmarinus officinalis* L.) containing carnosic and rosmarinic acid, together with the carnosol that are BACs associated with antifungal, anti-diabetic, antiulcerogenic, anti-inflammatory, antithrombotic, and antidepressant health properties [47,48]. Medicinal and aromatic plants extracts are generally recognized as safe (GRAS), thus can be used as natural substitutes for chemical additives [49]. For instance, sage (*Salvia officinalis* L.) has strong potential as additive for (functional) food production due to its antioxidant properties [50] and documented anti-inflammatory, anticancer, antimicrobial, and antiproliferative properties [50,51,52]. In oregano (*Origanum vulgare*), the main BACs are rosmarinic and caffeic acids with high amounts of flavonoids, namely hispidulin, and apigenin [53]. Consumption of oregano was associated with antimicrobial and antioxidant activity [54]. Thyme (*Thymus vulgaris* L.) mostly contains thymol, carvacrol, geraniol, and *p*-cymene [55] and shows antibacterial [56], respiratory [57], and neuroprotective properties [58]. Economic source of highly valued BACs is an industrial byproduct from winemaking, otherwise known as grape pomace, that has various polyphenolic antioxidants as flavonoids, phenolic acids, phenolic alcohols, stilbenes, and lignans [59]. They showed medicinal effects in atherosclerosis, hypertension, neurodegeneration, myocardial, and other diseases [59]. Similarly, citrus waste is commonly remained byproduct of citrus juice production that is rich with essential oils, limonoids, and polyphenols, e.g., flavonoids [60]. Citrus peel is particularly rich in flavonoids, carotenoids, dietary fiber, sugars, polyphenols, essential oils, and ascorbic acid [61]. BACs from citrus waste is mostly responsible for antioxidant activity while essential oils were antibacterial, antimycotic, antiviral, and antiprotozoal agents [62]. *Stevia rebaudiana* Bertoni leaves have a large potential for production of non-caloric sweeteners due to steviol glycosides and other by-products [63]. Indeed, stevia is also an excellent source of BACs such as polyphenols, chlorophylls, carotenoids, and ascorbic acid, while its extracts have antimicrobial, antioxidant [64,65], anti-hyperglycemic, anti-hypertensive, anti-inflammatory, anti-tumor, anti-diarrheal, diuretic, and immunomodulatory properties [66]. Olive leaves (*Olea europaea* L.) considered as both, waste from olive oil production and medicinal and aromatic plants have numerous polyphenols as oleuropeosides (oleuropein and verbascoside); flavonols (rutin); flavan-3-ols (catechin); flavones (luteolin-7-glucoside, apigenin-7-glucoside, diosmetin-7-glucoside, luteolin, and diosmetin). It also contains tyrosol, hydroxytyrosol, vanillin, vanillic acid, and caffeic acid [67,68,69,70]. Olive leaves extracts showed antitumor, antioxidant, hypolipidemic, antiviral, antimicrobial, cardiovascular, and anti-inflammatory health benefits [71,72,73,74,75,76]. Next step in obtaining high value-added compound is to identify the best extraction procedure and adequate processing relevant for food production.

Regardless of the method used to extract BACs, it is important to mention that the chemical composition should be assessed preferably using chromatographic methods (LC-MS, HPLC, and GC) and if the plant is not documented, toxicological studies using in vitro and in vivo assays should be carried out to attest its GRAS status. After that, possible functional effects in humans should be conducted, preferably using (pre)clinical trials, aiming to achieve a dose-dependent response.

## 3. Innovative Extractions and Processing of BACs

Any extraction procedure has a goal set to provide the highest amounts of target BAC in produced extract and with minimal contamination with other undesired compounds [12,77]. Hence, many extraction procedures are employed for such purpose. The solid–liquid conventional extraction is frequently used for extraction of polyphenols and other phytochemicals in order to facilitate the dissolution of the solutes, external transport by the release of solutes from a solid matrix to the solvent phase [4]. The steam and hydrodistillation are most common commercially employed conventional extraction for extraction of BACs from medicinal and aromatic plants [78]. The main extraction parameters are the type of extraction, plant material, particle size, solvent, acidity, temperature, time, and the addition of enzymes [79,80,81]. A great deal of BACs is thermally unstable and can be either degraded or completely lost during the production of conventional extracts. While the conventional extraction is economic, it is still a time-consuming process with low selectivity and large consumption of solvents [82,83], hence not aligned with principles of green chemistry [28,84].

In processing, storage, and transport, encapsulation shelters BACs from air exposure, humidity, and light. Hence, preserves nutritive value, bioavailability, solubility, and functionality of BACs. From food engineering point of view, encapsulation masks off-flavors and odors, deconcentrates BACs, controls their release, and stimulates their handling in foods [85].

### 3.1. Fruits

Food extraction research is focused on identifying processing technologies that have minimal influence on the native content of BACs in fruits [7]. Fresh fruits such as apple and kiwi have the tendency for browning and this can be partly alleviated by an ultrasound technology that can foster better penetration of antibrowning agents to fruit’s tissues [86,87]. Pasteurization and other thermal treatment of juices can reduce the sensory and nutritional quality of juices, hence there is growing interest for the development of nonthermal techniques for juice processing [25,26]. For instance, mandarin juices are susceptible to thermal degradation and loss of nutritive value, so for safe and high quality juices industry looked into nonthermal processing like pulsed electric fields (PEF), high pressure processing or high hydrostatic pressure (HPP/HHP), UV-light, pulsed-light, and irradiation treatments [13]. Stability of prickly pear fruit and BACs in processing and extraction are strongly affected by the type of used technology [12]. For this particular matrix, HPAE showed promising results for industrial applications. Conventional methods together with the HPAE and PEF showed promising potential for processing and for obtaining BACs from prickly pear, while supercritical CO_2_ extraction was effective for extraction of the oils [12]. Extraction of BACs from berries is commonly done by conventional extraction that include maceration, Soxhlet extraction, solid–liquid, and liquid–liquid extraction [18]. These approaches have (dis)advantages previously mentioned that are common for conventional techniques (e.g., economic but with consumption of time and solvents) and with detrimental repercussions on BACs in extracts due to thermal instability. Overall the conventional extraction from berries had larger yields, but innovative non-thermal extractions had the potential for lower costs of production, higher quality, and “green and sustainable approach” [88,89,90,91]. PEF showed good selectivity, while HVED provided more of total phenolics extracted from the grapes [92,93]. The UAE improved yields of total polyphenols and proteins as compared to controls, but overall results of extraction were less emphasized as compared to HVED and PEF.

### 3.2. Other Vegetables

Similar to other BACs from plants, organosulfur compounds (OSC) found in *Allium* sp. are thermally degradable compounds and regardless of the sterilization, pasteurization, drying, or cooking they tend to be lost with processing [16]. In this instance, increased temperature will have detrimental effects on enzymes relevant for bioavailability of OSC in *Allium* (i.e., allinase) as well as a direct negative effect on the content of the bioavailable OSCs (e.g., thiosulfinates). Freeze-drying [94] and infrared innovative drying [95] technologies limited the thermal degradation of the OSC, while boiling and autoclaving significantly induced the losses of OSC [96]. For garlic, the increased pressure during HPP decreased allinase activity and corresponding antimicrobial, antioxidative and anticancer properties [97]. Lower exerted pressures with HPP (300 MPa) improved pyruvate formation and enzymatic activity associated with OSC in garlic [98]. Similar was observed for pure allinase that lost its activity with increased pressure (from 150 to 300 MPa) [16]. OSC is mainly obtained for a nutraceuticals/food supplements by conventional and non-conventional methods. The OSC obtained by the CE were extracted for analytical purposes for evaluation of the biological activity [16]. The CE research targeted allicin and alliin from garlic cloves and plants but their stability in crude extracts was insufficiently documented [16]. From standpoint of innovative technologies, MAE and PLE were less suitable for OSC extraction, likely due to negative thermal effects, while SFE is an effective alternative for obtaining oleoresins and essential oils from *Allium* sp. However, the economic background should be correctly estimated for potential industrial purposes [16].

Carotenoids are one of the most interesting BACs found in pumpkin that can be lost with exposure to harsh thermal treatments [99]. HPP thermal treatments have the tendency to avoid carotenoid losses associated with conventional thermal processing together with the damages to the natural color in pumpkins [100]. Innovative technologies such as high pressure processing (HPP) and PEF have the tendency to preserve native contents of carotenoids in vegetables such as pumpkin and other foods [25,26,99]. Compounds that can be extracted from pumpkin are polysaccharides, pectin proteins, fixed oils, and sterols [101]. Polysaccharides are extracted from pulp with conventional extraction and with hot water and ethanol as solvents. Additionally, pectin is obtained from the same part of the plant but with the application of the enzymatic extraction. Extraction of proteins also employs conventional extraction similar to one for polysaccharides, but they are obtained from leaves and seeds. Oils from seeds are best obtained either by conventional extraction with a suitable solvent or with SFE procedures using CO_2_ as they limit thermal degradation and hydrolytic processes due to low aqueous phase [101]. Extraction of carotenoids is facilitated by high-pressure homogenization and their highest yields are obtained by conventional extraction [102]. However, as with other BACs this is a time consuming thermal method with the high expenditure of extraction solvents. SFE-CO2 with ethanol is reported as a good nonthermal method for carotenoid extraction. The UAE, PEF, and enzyme-assisted extraction are additional fast non-thermal alternatives to conventional extraction. Nevertheless, their economic background for industrial upscaling needs to be better evaluated in the literature [102].

### 3.3. Medicinal and Aromatic Plants

Similar to many other phytochemicals, BACs found in medicinal and aromatic plants are also thermally unstable and prone to degradation during processing. The most common conventional extraction used for commercial production of phytochemical extracts and essential oils is Soxhlet extraction that encompasses steam and hydrodistillation with Clevenger type apparatus [78]. Extracts of rosemary obtained by the conventional extraction and optimized by the response surface methodology (RSM) contained rosmarinic acid, carnosol, and carnosic acid from rosemary [103]. Antioxidant activity of sage conventional extracts was evaluated form two types of preparations, infusions and decoctions, where the later type had the highest polyphenolic content and antioxidant activity [104]. The conventional extraction of macerated dry medicinal and aromatic plants with hot water produced better extracts than those processed with cold water [105]. Extracts of sage, thyme, and oregano had rosmarinic, caffeic, cinnamic, chlorogenic, ferulic, quinic acids, in addition to apigenin, luteolin, and quercetin [106]. Extracts of thyme mainly contained polyphenols such as rosmarinic, caffeic, ferulic acids, and their derivatives [106]. The main BAC found in aqueous olive leaves extracts obtained by conventional extraction was oleuropein and its yields can be improved by pre-processing with air-drying [107].

Pretreatment with PEF of Mediterranean basil (*Ocimum basilicum*) positively affected retention of color, aroma, rehydration capacity in dried samples [108]. The encapsulation of BACs from medicinal plant extracts (e.g., raspberry leaf, hawthorn, ground ivy, yarrow, nettle, and olive leaf) with alginate–chitosan system produced microbeads with high polyphenol content and antioxidant activity [109]. Encapsulation of *H. perforatum* extract with β–cyclodextrin by freeze-drying improved the thermal stability of flavonoids [110]. Extraction of BACs from plants is lengthy process often accompanied by limited mass transfer and yields and with large consumption of toxic solvents. Hence, development of green extraction like Pressurized Liquid Extraction (PLE), UAE, PEF, and HVED for this purpose [111,112,113,114,115] will foster the development of green chemical engineering and sustainable production [28,116,117]. UAE with ethanol produced three times more of the rosemary extract than CE from dried extracts with high contents of rosmarinic and carnosic acid [118]. The similar observation was found for sage UAE extracts where this technology decreased the length of extraction and solvent consumption [119]. PLE with ethanol as solvent produced rosemary extracts rich with polar and non-polar antioxidants including carnosic and rosmarinic acids [27]. PLE proved specific, sensitive, and fast “green” extraction of oleuropein from olive leaves. The method produced high yields while depended on the affinity of subcritical SC-CO_2_ for nonpolar molecules [113]. PLE successfully extracted flavonoids, hydroxycinnamic acids, and flavonols from olive leaves and prove useful for industrial purposes [23]. Various extraction technologies were used for extraction of steviol glycosides from *Stevia,* they include electrotechnologies, MAE, UAE, PHWE, and supercritical CO_2_ [19]. Pressurized Hot Water Extraction (PHWE) is one of the promising “green extractions” able to provide safe extracts that are immediately ready for consumption. This extraction employs higher temperatures and pressure to maintain solvent in the liquid state, hence improving extraction and stability of polar and nonpolar BACs while being suitable for industrial scale-up [19].

### 3.4. Food Wastes and By-Products

Polyphenols and other natural antioxidants are frequently extracted from various low-cost raw material as berry waste (i.e., pomaces) by the conventional extraction [18]. Grape pomace serves as an economic source of polyphenols that are extracted by the 50% ethanol, so they can be later used for the production of nutraceuticals or functional foods. Polyphenols in conventional extraction are extracted with various percentage of ethanol in aqueous solutions for obtaining polyphenols with various polarities. However, longer exposure to elevated temperatures in conventional extraction will tend to degrade such compounds [22]. Aside from that, conventional extraction has some negative aspects in the use of toxic solvents (e.g., methanol acidified with hydrochloric acid, propanone, *n*-hexane etc.). Innovative technologies can be used to extract valuable BACs from pomace. A recent review reported main innovative technologies for the extraction of BACs from raw grapes, wastes and by-products with regards to food matrix, target BACs, and used technology [82]. HPAE outperformed UAE for 50% improvements in extraction efficiency for anthocyanins at 600 MPa [120]. Furthermore, anthocyanin monoglucosides were best obtained by the PEF vs. HPAE, however acylated anthocyanin was better extracted by the HPAE at 70 °C and 600 MPa with 50% ethanol [121]. Both studies did not report the cultivar types that might bias obtained findings [22]. A recent study reported that influence of temperature was greater than the influence of exerted pressure for obtaining the anthocyanins with HHPE [22]. Electroporation induced by PEF improved the selective release of intercellular pigments [92] while UAE improved extraction of total phenols, total anthocyanins and total proteins [122] from wine grapes. One of the reviews gave an overview of extraction technologies for obtaining the BACs from the raw grapes, wastes, and byproducts and reported further need to optimize processing parameters with regards to natural sources, BACs, and used technology [82]. 

A recent review gave the thorough report about extraction of the BACs from citrus waste [4]. Citrus waste is an industrial source of pectin that is obtained by conventional extraction at acidic pH and elevated temperatures. Further steps include precipitation by alcohol and subsequent purification [123]. Polyphenols extracted from citrus waste are obtained by conventional extraction at temperatures from 50–100 °C [4]. Limonene, γ-terpinene, and *p*-cymene re-obtained by steam distillation and cold-press systems where latter proved to be a better option for extraction. MAE [124,125,126], UAE [126,127], HPAE [128,129], subcritical water [130], and others [4] were used as innovative alternatives to CE of pectin from citrus waste. Best yields were obtained by MAE and addition of water where processing with five times increase in yield with freeze-drying. Extraction of polyphenols such as naringin and hesperidin from citrus waste increased with PEF pre-processing with the increase of electric strength and treatment time, likely due to the effect of electroporation [4]. UAE outperformed conventional extraction of flavonoids at the lower temperature and in shorter times [131]. Different research showed that not all flavonoids were stable with UAE treatment where eriocitrin, narirutin, neohesperidin, quercitrin, eridictyol, didymin, naringenin, luteolin, sinensetin, nobiletin, tangeretin, naringin, and hesperidin retained stability during UAE while quercetin degraded [132]. MAE was confirmed to be as a reliable method with the minimum expenditure of solvent for extraction of BACs from citrus peel [4]. HPAE more successfully extracted polyphenols from orange and lemon peels in comparison to controls [133,134]. Spray drying was processing technology able to produce powders with rich with antioxidant BACs from citrus waste. Hesperidin and cyanidin 3-glucoside were the main BACs with good antioxidant activity in the spray-dried citrus by-products [4]. SC-CO_2_ extraction was better than conventional extraction for extraction of flavonoids from pomelo waste in terms of improved yield obtained in a shorter time and with better antioxidant activity [135].

## 4. Conclusions and Future Prospects and Challenges

As BACs include an extremely diverse class of compounds (polyphenols, tocopherols, organosulfur compounds, carotenoids, etc.) with different chemical structures (hydrophilic or lipophilic), various distribution in nature (specific to fruit, vegetable or medicinal plant species or ubiquitous), wide range of concentrations, and biological action, it is necessary to find an optimal extraction procedure that will efficiently extract the target compounds in desired concentrations with the highest biological value. Therefore, the selection of particular technique significantly depends of the targeted BACs, and in both short & long-term future should involve the green extraction concept and principles [136]. A number of novel food processing and preservation procedures have been established and applied even for industrial purpose in order to avoid unfavorable nutritional and sensory changes during the conventional heat treatment [137]. However, with the aim to increase production and process efficiency with minimal or no changes in nutritional and biological properties of foods, reduce solvent and energy consumption and decrease food waste by improving shelf life, green chemistry has an important impact in changing industrial and academic practices [138]. Therefore, efforts are made on developing more environmentally sustainable production systems in order to develop safe and high-quality green products.

Sources of BACs are abundantly found in medicinal plants, fruits, vegetables and, economically, in wastes that often remains after food processing. Such wastes represent sustainable raw materials that are able to provide nutritive, economic, and eco-friendly production of functional foods. The extraction conditions and processing greatly depend on the source and type of BAC, and applied technology. All relevant extraction parameters should be mathematically optimized according to the strict scientific methods in order to obtain the most economical production with highest-quality extracts. Based on the BAC polarity, some extractions are better than the others, so for polar BACs supercritical CO_2_ extraction provides very good results, while for others and less polar BACs accelerated solvent extraction or pressurized hot water extraction are very good options. In many cases, UAE seems as good pretreatment method for further processing.

Food production is gaining momentum on global food markets and there are industrial requirements to perfect extraction technologies for obtaining high-quality extracts that can be used as raw materials for its production. Even though conventional extraction still is the main approach for obtaining BACs, this technology is not aligned with “green” and sustainable production, as it is often accompanied by high expenditure and disposal of toxic chemicals and energy. Moreover, this thermal technology tends to damage thermally unstable BACs that supposed be produced in the first place. On the other hand, innovative options are often more selective, faster, sustainable, thermally sensitive, but still not sufficiently tested for industrial purposes. Plenty of various arguments label them as expensive, and impractical for industrial applications, due to the expensiveness of the industrial equipment that for some technologies is at the prototype level or has to be tailor-made for each particular application. It is very promising that the European Commission and its Research Executive Agency provide funding for implementation of such innovative solutions in the industry by Horizon 2020 work programs (e.g., SFS-16-2018). Hence, in closing, future food manufacturing can foresee better solutions for industrial production and applications.
